# Emergency Department Initiative to Decrease High-flow Nasal Cannula Use for Admitted Patients with Bronchiolitis

**DOI:** 10.1097/pq9.0000000000000728

**Published:** 2024-05-09

**Authors:** Courtney E. Nelson, Jonathan M. Miller, Chalanda Jones, Emily Reese Fingado, Ann-Marie Baker, Julie Fausnaugh, Michael Treut, Leah Graham, Katlyn L. Burr, Arezoo Zomorrodi

**Affiliations:** * From the Department of Pediatrics, Nemours Children’s Health, Wilmington, Del.; † Respiratory Care Department, Nemours Children’s Health, Wilmington, Del.

## Abstract

**Background:**

Despite limited evidence, a high-flow nasal cannula (HFNC) is often used to treat mild to moderate (m/m) bronchiolitis. We aimed to decrease the rate of HFNC use in the pediatric emergency department (PED) for m/m bronchiolitis from a baseline of 37% to less than 18.5%.

**Methods:**

A multidisciplinary team created a bronchiolitis pathway and implemented it in December 2019. A respiratory score (RS) in the electronic medical record objectively classified bronchiolitis severity as mild, moderate, or severe. We tracked HFNC utilization in the PED among patients with m/m bronchiolitis as our primary outcome measure between December 2019 and December 2021. We monitored the percentage of patients with an RS as a process measure. Interventions through four plan-do-study-act cycles included updating the hospital oxygen therapy policy, applying the RS to all patients in respiratory distress, modifying the bronchiolitis order set, and developing a bronchiolitis-specific HFNC order.

**Results:**

Three hundred twenty-five patients were admitted from the PED with m/m bronchiolitis during the 11-month baseline period and 600 patients during the 25-month intervention period. The mean rate of HFNC utilization decreased from 37% to 17%. Despite a decrease in bronchiolitis encounters after the pandemic, in the spring of 2021, when volumes returned, we had a sustained HFNC utilization rate of 17%. RS entry increased from 60% to 73% in the intervention period.

**Conclusions:**

A clinical pathway for bronchiolitis can lead to decreased use of HFNC for m/m bronchiolitis. Consistent RS, order set development with decision support, and education led to sustained improvement despite pandemic-related volumes.

## INTRODUCTION

Bronchiolitis is the leading cause of hospitalization in children younger than 1 year, accounting for approximately 100,000 hospital admissions and US healthcare spending of over $1.7 billion annually.^1,2^ Despite decreased bronchiolitis hospitalizations over the past decade, overall admission costs have increased without a correlating increase in illness severity.^3^ One contributing factor could be the increasing use of high-flow nasal cannula (HFNC) therapy, which has become more widely adopted during this period.^2,3^

In 2014, the American Academy of Pediatrics published a clinical practice guideline for diagnosing and treating bronchiolitis, emphasizing supportive therapies such as supplemental oxygen and fluid administration.^4^ At the time of publication, there was limited evidence on the efficacy of HFNC, and therefore, there was no recommendation for or against its use.

HFNC has not been shown to impact the clinical course for patients with mild to moderate (m/m) respiratory distress; however, it may decrease progression to invasive ventilation in patients with severe illness.^5–7^ Despite limited evidence of its utility in m/m bronchiolitis, HFNC remains a popular intervention for bronchiolitis.^8^ Overutilization of HFNC leads to increased pediatric intensive care unit (PICU) admissions and hospital length of stay (LOS) without clinical benefit.^6,9^ Using respiratory scores (RSs) to assess clinical severity objectively allows for appropriate resource allocation and evaluation of response to treatment.^10–12^ There are several validated scoring tools, though no tool has been universally recommended.^13–15^ A large multicentre study found that protocols to limit HFNC decreased the odds of initiating HFNC and reduced the duration of HFNC and hospital LOS.^16^ At our institution, HFNC is often initiated in the pediatric emergency department for patients with m/m disease, with significant variability among providers. Before our work, HFNC was initiated in 37% of patients admitted for m/m bronchiolitis, and our average hospital LOS was 41.4 hours. We aimed to reduce the initiation of HFNC in the emergency department (ED) for patients admitted with m/m bronchiolitis by 50%, from a baseline of 37%–18.5% over 6 months.

## METHODS

### Context

Our 208-bed free-standing children’s hospital houses a 42-bed ED that sees approximately 60,000 patients and admits approximately 12,000 patients annually. Historically, 10% of admissions are for bronchiolitis. The ED is staffed by categorical pediatric residents, physician assistants, pediatric emergency medicine fellows and attendings, and visiting residents from emergency medicine, family medicine, and other pediatric residency programs. Our nursing staff includes one nurse for every four patient rooms, two triage nurses, and one additional float nurse. We have two respiratory therapists from 11 am to 11 pm and one from 11 pm to 11 am. At the time of our intervention, there was an ED bronchiolitis pathway and a bronchiolitis scoring tool primarily used by the nursing and respiratory staff. HFNC was used on the general pediatric floor with a minimum setting of 4 L per min and a maximum setting of 10 L per min for patients under 2 years of age. All patients on HFNC requiring higher settings were admitted to the PICU. An oxygen therapy policy addressed the general use of oxygen therapy but was not bronchiolitis-specific.

### Study Population

We included patients aged 1 month to 2 years presenting to the emergency department (ED) with lower respiratory tract illness exhibited by tachypnea, wheezing, or adventitious breath sounds. Exclusion criteria included patients directly admitted to the PICU and/or intubated in the ED, patients with asthma, and patients with a “medically complex” diagnosis in their chart because they may not respond to standard bronchiolitis management. Medical Complexity is defined using an EMR scoring tool (CRG = 3M Clinical Risk Groups) as well as clinically, including children with one or more chronic health conditions associated with significant morbidity and mortality, impact on acts of daily living, high healthcare utilization, including the need for three or more specialties, and dependence on technology. Finally, we excluded all transports to the ED because their initial management was impacted by the care provided at the outside hospital. Patients with prematurity alone, nonasthmatics with some response to albuterol, and those with bronchiolitis and secondary pneumonia were included.

### Interventions

**Fig. 1.**
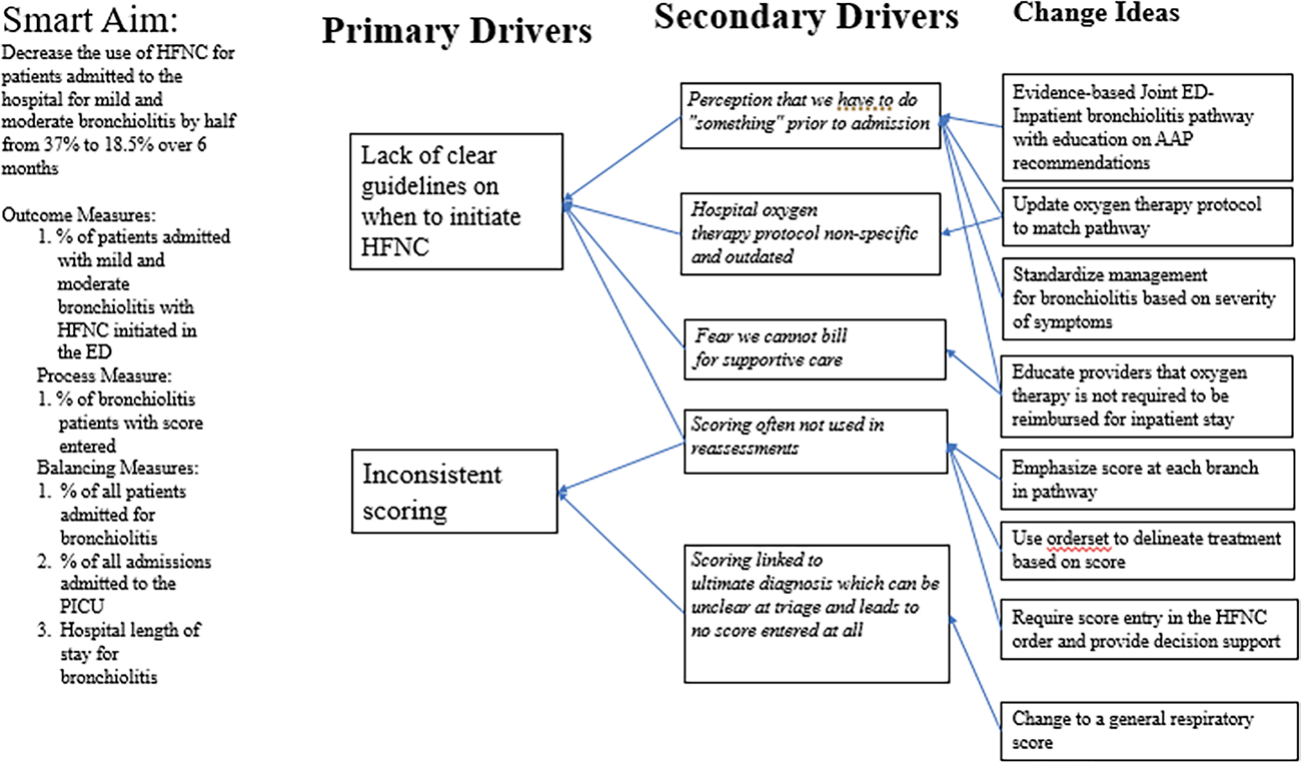
Key driver diagram.

In the spring of 2019, a multidisciplinary team was created to improve the value of care by decreasing the inappropriate use of HFNC to treat bronchiolitis. Team members included attending providers, respiratory therapists, and nursing leadership from the ED and inpatient services. We identified two key drivers of our current process: inconsistent use of the bronchiolitis scoring tool and lack of uniform indications for HFNC initiation. Secondarily, providers believed they could not bill for supportive care alone and felt pressure to “do something” before admission (Fig. 1).

### Pathway Development

We created a combined ED and inpatient bronchiolitis pathway to improve consistency across both departments. To emphasize the importance of scoring, we assigned a bronchiolitis score as the first step of the pathway. Patients were categorized as mild (score 1–4), moderate (score 5–8), or severe (score 9–12), and each category had an associated treatment algorithm.^17^

**Fig. 2.**
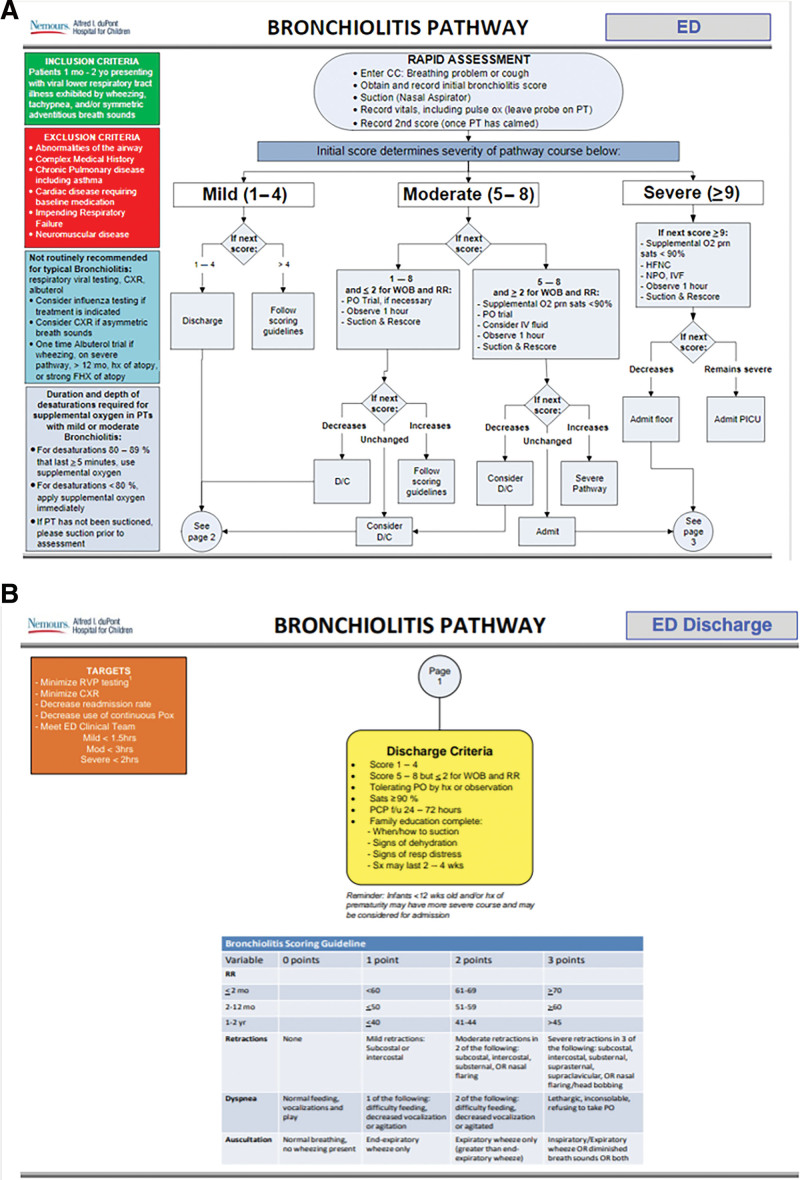
A–B, Final combined ED-inpatient bronchiolitis pathway. Each patient is assigned a respiratory score, which determines the treatment branch they receive. *The “if next score” decision point indicates the score after suctioning.* This is the first section of the combined pathway: the ED section. *The discharge box specifies the criteria for discharge.* PT, patient; WOB, work of breathing; RR, respiratory rate; PO, per os; NPO, nil per os; IVF, intravenous fluids; D/C, discharge.

Scoring was done consecutively by respiratory therapists, nurses, and providers. Emphasis was placed on repeating a score postsuctioning. HFNC was only recommended for those patients with severe bronchiolitis scores who failed to improve with suctioning or those with m/m scores who progressed to severe scores, with decision-making about the initiation of HFNC shared between respiratory therapists and the provider team. Scoring was repeated after HFNC initiation. Patients were only eligible for floor admission if their score improved to moderate severity on HFNC rates less than or equal to 12 L per minute; otherwise, they were admitted to the PICU. This increased from the prior practice of allowing flow rates less than or equal to 10 L per min on the pediatric floor. This recommendation was reached by consensus given that most of our patients under 2 years of age would receive less than 2 L per kg per minute, a common threshold for PICU admission at other institutions, on 12 L per min. Our coding team confirmed that oxygen therapy was not required for admission billing. Therefore, the pathway recommended admission for supportive care alone for patients with m/m bronchiolitis who presented with a combination of retractions and tachypnea that failed to improve with suctioning (Fig. 2A,B). Low-flow oxygen was limited only to those patients with hypoxemia (<90%) who did not meet the criteria for HFNC.

### EMR Integration

Before pathway initiation, we developed ED and inpatient bronchiolitis order sets in the electronic medical record (EMR) with order groupings specific to illness severity. There was a nursing order for frequent suctioning and repeat scoring for all patient severities. The HFNC order was only found under the severe category.

#### Plan Do Study Act Cycles

Pathway education began in October 2019. The pathway was implemented on December 9, 2019. Process and outcome data were reviewed every 30 days for the first 90 days postimplementation. After each 30-day data review, the pathway leader for ED respiratory therapy, nursing, and physicians presented the data to their respective groups, re-educated on the aims of our work and specifics of the pathway, and solicited feedback. The pathway group reconvened and identified key process failures after the data review. First, if the timing of the score entry was not close to the initiation of HFNC, it could misrepresent the patient’s severity during HFNC initiation. For this, providers were re-educated to enter a score before HFNC initiation. Second, diagnostic uncertainty related to the overlapping symptoms of asthma and bronchiolitis, which had separate scoring tools, led to variable scoring because providers could not decide which scoring tool to use. Finally, we found that the hospital oxygen therapy protocol did not specify that a patient with bronchiolitis should be in severe distress before the initiation of HFNC. We re-educated our respective groups that the pathway document should drive treatment and that the oxygen therapy policy is not specific to bronchiolitis. When our educational efforts were not impactful, we began to discuss more automated and standardized interventions, plan do study act (PDSA) 1: modified oxygen therapy protocol; PDSA 2: creation of a new bronchiolitis order set, and PDSA 3: changing the name of the bronchiolitis score to a “respiratory score” and using it for both asthma and bronchiolitis. However, these efforts were interrupted by the COVID-19 pandemic in early 2020.

In the summer of 2020, regular pathway meetings resumed. The oxygen therapy protocol (PDSA 1) was updated to specify that HFNC for bronchiolitis should be reserved for patients with severe respiratory distress and weaned once respiratory distress improves.

In the fall of 2020, a fourth data review found that the score’s timeliness relative to the HFNC order placement still needed to be improved. In March 2021, we modified the ED bronchiolitis order set (PDSA 2) to require the entry of a respiratory score before ordering HFNC. We added the following decision support: “HFNC is only recommended for patients with a respiratory score of 9 or more.” The order could still be placed if the score entered was not severe. Finally, in the summer of 2021, the bronchiolitis score was adapted to include patients over 2 years of age and re-named the “respiratory score” (PDSA 3). The new respiratory score was identical to the old bronchiolitis score for patients with bronchiolitis. Providers were instructed to use this respiratory score for all respiratory complaints to mitigate the lack of score entry due to an unclear diagnosis.

In the fall of 2021, when we saw an increase in patients with bronchiolitis, a fifth data review found that providers were still ordering HFNC outside the bronchiolitis order set. In response, a bronchiolitis-specific HFNC order (PDSA 4) with score entry and decision support was developed in November 2021 that populated as a preferred order when HFNC was searched in the ordering screen.

#### Measures/Analysis

The primary outcome measure was the percent of patients admitted with m/m bronchiolitis with HFNC initiation in the ED. Disease severity was categorized based on the most severe score documented, not the first score documented, to capture those patients that clinically worsened during their ED stay. The process measure was the percent of all patients seen for bronchiolitis in the ED with at least one documented bronchiolitis score and, after score adaptation, a respiratory score. Balancing measures included the proportion of all patients seen in the ED for bronchiolitis admitted to the hospital, the proportion of all bronchiolitis admissions admitted to the PICU at any point in their stay and the mean hospital LOS for bronchiolitis.

An electronic dashboard for the ED extracted EMR data for patients who met our inclusion criteria and tracked their admission rates, bronchiolitis scores, and HFNC use in addition to other treatment data. During the dashboard development, the team members validated the dashboard with an EMR review for each data element. De-identified patient data were extracted for all PDSA cycles and data review.

All outcome and process measures were tracked using p charts. No patients were admitted in May or June of 2020, and several months had fewer than 10 patients that met inclusion criteria, so outcome data were organized into groups of 25 encounters. Bronchiolitis scoring data were grouped into 75 encounters to stay under the limit of 80 control chart data points. Standard rules were applied to determine if changes were due to common or special cause variation.^18^ Baseline points that were out of the control limits were ghosted in the analysis of the baseline means. Statistical process control charts were created using QI-charts software, v.2.0.23 (Scoville Associates, 2009) for Microsoft Excel (Microsoft, 2016).

#### Ethical Considerations

This project was deemed exempt from our institutional review board’s approval.

## RESULTS

**Fig. 3.**
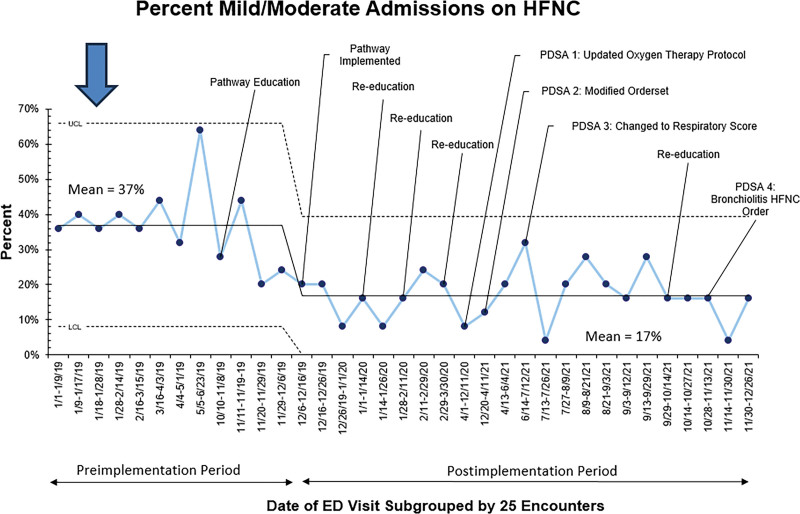
Outcome measure: Percent of patients admitted with mild and moderate bronchiolitis placed on HFNC. Data were grouped into 25 encounters per data point. The baseline point for 25 admissions between June 26, 2019 and October 7, 2019 was ghosted because the point was outside the control limits. UCL, upper confidence limit; CL, confidence limit.

In the baseline period from January to November 2019, 325 patients were admitted with m/m bronchiolitis. In the postimplementation period from December 2019 to December 2021, 600 patients were admitted with m/m bronchiolitis. There was no difference in the percentage of patients scoring mild, moderate, or severe disease between the baseline and postimplementation periods (*P* = 0.41). Admission on HFNC decreased from 37% preimplementation to 17% postimplementation (Fig. 3). A downward trend was noted following pathway education in November 2019. Special cause variation was identified in the first 25 encounters postimplementation, with a run of 8 points below the baseline and a new mean of 17%. During the next 18 months, the rate of HFNC use among those admitted for m/m bronchiolitis was variable but did not meet special cause variation. Once volumes increased in May and June of 2021, the percentage of patients admitted with m/m bronchiolitis on HFNC remained at 17%.

**Fig. 4.**
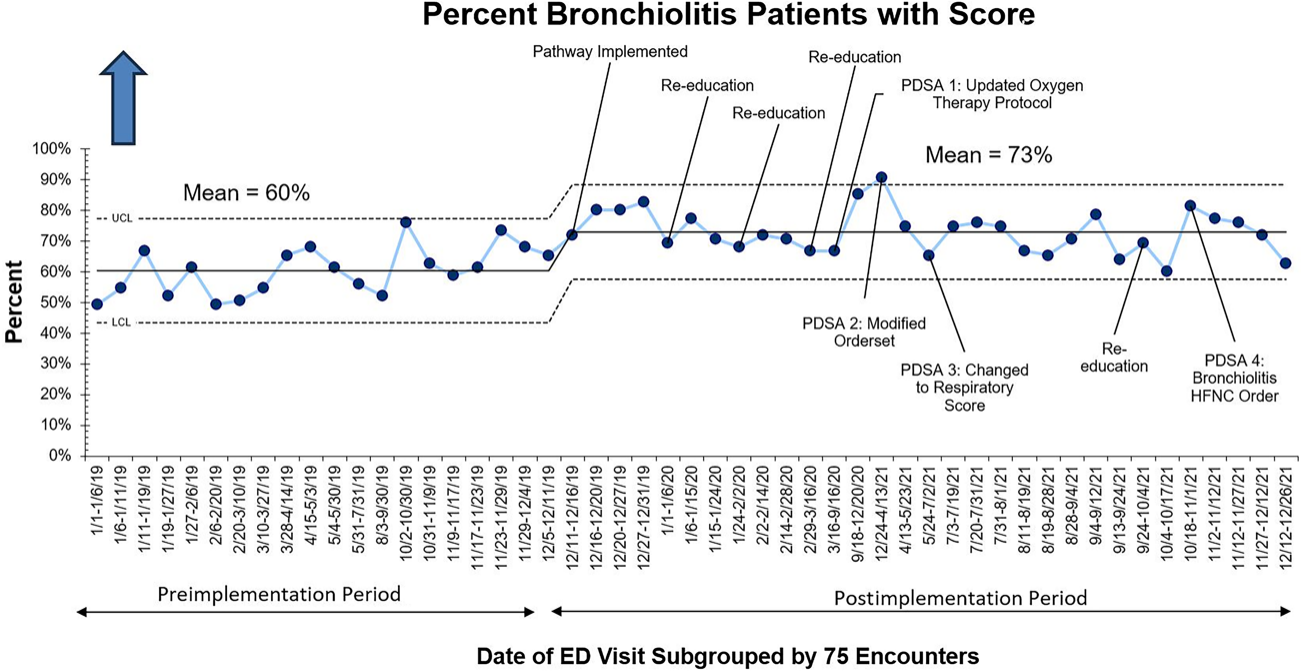
Process measure: percent of patients seen in the ED for bronchiolitis with a respiratory score documented. Data were grouped into 75 encounters per data point to meet the maximum recommended number of data points of 80. UCL, upper confidence limit; LCL, Lower confidence limit.

A bronchiolitis score was documented in 60% of patients with bronchiolitis in the preimplementation period (Fig. 4). This increased to an average of 73% immediately following pathway implementation. Despite a common cause downward trend in scoring rates in the spring of 2021, initiating a universal respiratory score reverted this trend, sustained through the fall of 2021.

The proportion of all patients with bronchiolitis admitted to the hospital did not change postpathway implementation (38% versus 35%, *P* = 0.58). However, of those admitted, the proportion of patients admitted to the PICU decreased after implementation from 7.4% to 4.4% (*P* = 0.02). When we extrapolated the preimplementation PICU admission rate to the total population admitted postimplementation, the reduced PICU admission rate correlated to 26 patients, avoiding a PICU admission. The pathway did not significantly impact hospital LOS (41.4 hours preimplementation versus 42.9 hours postimplementation, *P* = 0.41).

## DISCUSSION

Implementing a combined ED-inpatient bronchiolitis pathway reduced the initiation of HFNC in the pediatric emergency department for patients admitted with m/m bronchiolitis from 37% to 17%. Respiratory score documentation increased from 60% to 73%. While the balancing measures of admission rate and hospital LOS were not significantly different postimplementation, PICU admissions decreased.

Standardized clinical pathways for acute bronchiolitis have been shown to promote high-value care. Prior studies have focused on standardizing HFNC initiation and weaning, whereas others have focused on using HFNC on the pediatric floor instead of the PICU.^19–23^ Jo et al published similar work with an HFNC initiation protocol. However, their outcome measure was a reduction in HFNC for all bronchiolitis admissions, whereas our pathway specifically targeted patients admitted with m/m disease.^24^ Another study demonstrated a reduction in the use of HFNC by trialing low-flow oxygen for breathing work first, and only if that failed was HFNC initiated.^25^ However, low-flow oxygen reinforces the “do something” mentality that can lead to overuse of resources. Our pathway emphasized suctioning and supportive care for m/m bronchiolitis patients. Prior work has also spanned relatively similar respiratory seasons with expected variability in patient volume. Our work came at a unique time where we could implement our intervention during a standard respiratory season and then monitor its sustainability through the COVID-19 pandemic when we saw unprecedented low patient volumes followed by unseasonal increases in bronchiolitis.

This study found no difference in admission rate or hospital LOS, similar to prior studies.^26,27^ Tarantino et al found that after implementing a bronchiolitis pathway with HFNC initiation and weaning guidelines, HFNC duration decreased without changing hospital LOS or escalation of care.^28^ This finding conflicts with other studies that show that a decrease in HFNC treatment leads to a decrease in hospital LOS.^29^ One explanation could be that HFNC has no impact on the course of bronchiolitis for m/m disease and, therefore, has no impact on LOS. Alternatively, the greatest benefit of decreased LOS may be seen in those with severe disease alone.

This study demonstrated a decrease in PICU admissions postimplementation. Similarly, Coon et al showed that HFNC on the ward leads to increased use of PICU beds.^9^ We hypothesize that when HFNC is used in m/m bronchiolitis, the patients fail to improve, and the flow rate is titrated to maximum settings, which prompts a PICU admission. The maximum HFNC flow rate allowed on the floor did increase with the new pathway from 10 L per min to 12 L per min, which could have mitigated some PICU admissions. However, only 6 of the 600 patients admitted to the floor in the postimplementation group had a maximum flow rate of 12 L per min, suggesting that this change in the pathway had little impact on PICU admissions.

The highlight of this quality improvement project is its sustainability despite the dramatic decline in bronchiolitis admissions following the COVID-19 pandemic. During this unprecedented time, there was concern that clinical standard work would suffer. In April 2020, a few patients were admitted with bronchiolitis, with an increase in the percentage of HFNC use. The uncertainty of the COVID-19 disease process may have triggered this finding. However, following 12 months with few to no admissions for bronchiolitis and minimal use of the pathway, there was an unseasonal increase in bronchiolitis cases. This increase in visits is correlated with reduced HFNC use for m/m disease. This sustainability is a testament to the collaboration within our multidisciplinary team. We noticed this increase in cases and timed our fifth PDSA with data review and re-education. This collaboration proved crucial in higher-than-normal staff turnover and severely altered seasonal viral patterns. In addition, the modifications to the order set, including embedded decision support and the transition to a universal scoring tool just before the surge in cases, standardized the care for bronchiolitis patients regardless of volume.

This study was conducted at a single site, which may limit generalizability; however, we believe this workflow is translatable to other institutions. Another limitation of the study is that the pathway depends on score entry. Because we never achieved 100% score entry, some patients could have escalated to HFNC without a score demonstrating the heightened severity. However, 95% of patients admitted on HFNC postimplementation had a documented respiratory score. Additionally, providers could modify their scores to support the clinical management they were providing. However, we found no difference in the percent of patients scoring mild, moderate, or severe postpathway implementation (*P* = 0.41) despite a decline in HFNC use among those with m/m disease. We also tried to mitigate score alterations by having the respiratory therapist and provider jointly decide to initiate HFNC. Lastly, the EHR did not prevent providers from ordering HFNC if a patient had m/m disease severity. We felt such restrictions would disengage providers from standardization altogether, but this may be a worthwhile endeavor.

## CONCLUSIONS

A combined ED-inpatient bronchiolitis clinical pathway with decision support regarding the initiation of HFNC can significantly decrease inappropriate utilization of HFNC for patients with m/m bronchiolitis. Key interventions included consistently using a single respiratory score tool, order set development with embedded decision support, and increased provider awareness about the lack of evidence supporting HFNC use. Despite changes in volume and seasonality driven by the COVID-19 pandemic, we noted sustained improvement. Further research should determine the utility and impact of HFNC for m/m bronchiolitis patients.
